# Occurrence and detection method evaluation of group B streptococcus from prenatal vaginal specimen in Northwest China

**DOI:** 10.1186/s13000-016-0463-9

**Published:** 2016-01-20

**Authors:** Yun Xie, JunLan Yang, Peng Zhao, Hui Jia, Qi Wang

**Affiliations:** Department of Clinical Microbiology, Medical Laboratory Centre, Northwest Women’s and Children’s Hospital, Xi’an, Shaanxi 710061 China; Colleges of Life Sciences, Northwest University, Xi’an, Shaanxi 710069 China; Department of Clinical Laboratory, the Second Affiliated Hospital of Xi’an Jiaotong University, Xi’an, Shaanxi 710004 China

**Keywords:** Group B *streptococcus*, Pregnant women, Colonization rate, Carrot Broth, PathoDxtra Strep Grouping Kit, Direct latex agglutination test

## Abstract

Sensitive and efficient detection of Group B *Streptococcus* (GBS) colonization in pregnant women is essential for prescription of prophylaxis at the time of delivery as GBS is an opportunistic pathogen known to cause infant mortality. In this report, two studies were conducted on the methods of GBS detection in Shaanxi province, China, a region lacking data for GBS detection and occurrence. For Study 1, 100 GBS culture-positive vaginal swabs were collected from 1,567 pregnant women for evaluation by direct latex agglutination test. In Study 2, 200 GBS vaginal swabs were evaluated by three culture methods (sheep blood agar (SBA), Columbia colistin-nalidixic agar (CNA), and selective carrot broth (SCB)) followed by analysis using a latex agglutination test. GBS was detected in 6.4 % of specimens in Study 1 and 10.5 % of specimens in Study 2. The results of the latex agglutination test in both studies were accurate with samples exhibiting high to moderate GBS growth, but the accuracy declined for samples with low GBS growth. The evaluation of culture methods for GBS detection revealed the sensitivity of SCB (95.2 %, *p* = 0.004) was significantly higher than that of the SBA medium (57.1 %). The sensitivity reported for SCB (95.2 %) was higher than CNA (76.0 %), but the difference was not statistically significant *(p* =0.078). These results indicate a selective broth, such as SCB, is ideal for accuracy at low growth levels, but a direct latex agglutination test could be used as an alternative for rapid detection of GBS in circumstances requiring immediate detection.

## Findings

### Introduction

Group B *streptococcus* (GBS) is a pathogenic bacterium that causes morbidity and mortality among neonates worldwide [10, 21]. Vertical transmission of GBS from a puerperant to her newborn during delivery can lead to GBS infection resulting in a fatality rate of approximately 50 % [5]. The rate of neonatal GBS infection ranges from 0.17 to 3.06 per 1000 live births in developing countries [4, 8].

Studies have shown that GBS screening and intrapartum antibiotic prophylaxis can prevent neonatal transmission of GBS [18]. The sensitivities of detection methods for perinatal GBS colonization are, however, dependent upon specimen delivery time and culture medium [15]. Traditionally, specimens were evaluated using two solid medium, sheep blood agar (SBA) and Columbia colistin-nalidixic agar (CNA), both of which underestimate the incidence of GBS [12]. Therefore, the CDC recommends use of a selective broth medium, such as LIM broth or carrot broth (SCB) [22]. Previous studies revealed the colonization rate of GBS using the SCB culture method was up to 15 % more sensitive than LIM broth ([7, 19]). However, this method is expensive and time-consuming as it takes at least 6 hours to observe a positive reaction [2]. Therefore, evaluation of current methods to determine the most sensitive, cost effective and time efficient detection test is beneficial [16]. The aim of this study was to evaluate the occurrence and detection methods of GBS from pregnant women in Shaanxi province, China.

## Materials and methods

### Study design

This research, consisting of two independent studies, was conducted at Northwest Women’s and Children’s Hospital in Xi’an, China. For Study 1, 1,567 pregnant women at 32–37 weeks of gestation were selected from January 2014-December 2014 for GBS screening by the SBA method followed by a direct latex agglutination test. For Study 2, 200 pregnant women were selected from December 2014-March 2015 for GBS screening by the SBA, CNA and SCB culture methods followed by a latex agglutination test. All individuals that recently used antibiotics, had symptomatic vaginal discharge, or an acute illness were excluded. The Research Ethics Committee of Northwest Women's and Children’s Hospital approved the study and gave the approval number (#3/2014) and informed written consent was obtained for all participants.

### SBA, CNA, and SCB methods

**Fig. 1:**
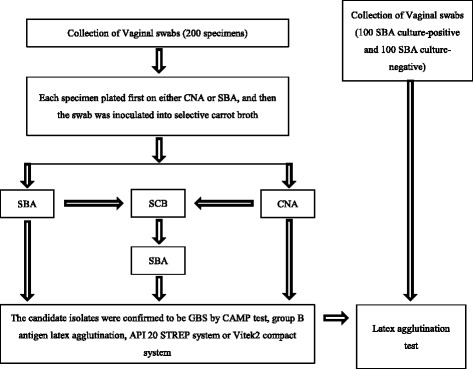
Laboratory procedure of the vaginal specimens for the isolation and latex agglutination test of GBS

Duplicate vaginal swabs were collected simultaneously from each pregnant woman for both studies using the Copan ESwab Collection and Transport System (Copan Italia, Brescia, Italy). Of the duplicated swabs, one swab was plated on either SBA only (for Study 1) or on either CNA (Becton, Dickinson and Company, Shanghai, China) and SBA followed by culture in SCB (Hardy Diagnostics, Santa Maria, CA) for Study 2. The other swab was stored at 4 °C. All CNA and SBA plates were incubated at 37 °C and 5 % CO_2_ for 18 to 48 h and all SCB cultures were incubated at 35 °C ambient air for 18 to 48 h. Aliquots of SCB cultures were subsequently plated on SBA. Cultures growing in SCB were positive if a visible change from colorless to red/orange was observed. Confirmation of GBS-like isolates was achieved by either the CAMP test, latex agglutination test (PathoDxtra Strep Grouping Kit), API 20 STREP system, or Vitek2 compact system prior to an additional latex agglutination test (Fig. 1).

### Latex agglutination test method

Analysis of GBS positive specimens determined by SCB was achieved using 0.5 mL of culture in a direct latex agglutination test. Alternatively, analysis of GBS positive specimens by the SBA and CNA culture method was achieved using extracts from the corresponding refrigerated swab. The PathoDxtra Strep Grouping Kit (Oxoid) was used to extract and/or test the samples according to manufacturer’s instructions. Each latex agglutination test included one negative control swab immersed in sterile broth and one positive control swab dipped in a broth containing group B strain ATCC 12403. The turbidity of the suspension was adjusted to the McFarland 0.5 turbidity standard. All samples were stored at 4 °C.

### PCR analysis

Polymerase chain reaction (PCR) was used to confirm positive growth of GBS from samples in Study 2 by detection of the *cfb* gene (CAMP factor). PCR primers for *cbf* were as follows: primer 1 (5′-ATC GTT ATG GTT TTT ACA TGA-3′) and primer 2 (5′-TTA TTT TAA TGC TGT TTG AAG TG-3′). Parameters for PCR amplification were as previously described [1].

### Statistical analysis

The significance test of proportions was used to determine if detection rate differences were significant. The McNemar test for correlated percentages and the χ^2^ test with Yates’ correction were used to analyze qualitative variables. The alpha level was set at 0.05, in which *p* < 0.05 was considered statistically significant.

## Results

### Latex agglutination test result of SBA method

In Study 1, the median age of enrolled women was 25.7 ± 4.8 years. Out of 1,567 samples analyzed, 6.4 % (100/1,567) were positive. The positive vaginal swabs determined by the SBA method were categorized into the following four levels of growth density: high (>100 c.f.u. per swab), moderate (50–100 c.f.u. per swab), low (10–50 c.f.u. per swab) and little (<10 c.f.u. per swab). Of the 100 culture-positive samples, 75 % were found to be GBS positive by direct latex agglutination (Table 1). Cultures with heavy or moderate density yielded robust results of 100 % for sensitivity, specificity, positive predictive value (PPV), and negative predictive value (NPV). The sensitivity and negative predictive value declined considerably with density, 66.7 % and 75 % for low density and 13.3 % and 53.6 % for little density, respectively. This was a screen test. It is not necessary to do statistical analysis.

**Table 1:** Latex agglutination test results as compared with different density levels of GBS for 100 culture-positive specimens

Density level of GBS (c.f.u. per swab)	No. of samples tested positive (*n*)	No. of samples positive for GBS (*n*)	Sensitivity (%)	Specificity (%)	PPV (%)	NPV (%)
High (>100)	22	22	22/22 (100 %)	22/22 (100 %)	22/22 (100 %)	22/22 (100 %)
Moderate (50–100)	27	27	27/27 (100 %)	27/27 (100 %)	27/27 (100 %)	27/27 (100 %)
Low (10–50)	24	36	24/36 (66.7 %)	36/36 (100 %)	24/24 (100 %)	36/48 (75.0 %)
Little (<10)	2	15	2/15 (13.3 %)	15/15 (100 %)	2/2 (100 %)	15/28 (53.6 %)

### SBA, CNA, and SCB followed by latex agglutination test

A total of 200 vaginal swab specimens were collected for evaluation of GBS detection by SBA, CNA, and SCB in Study 2. The median age of enrolled women was 24.3 ± 4.6 years. Of the 200 specimens tested, 10.5 % (21/200) tested positive by PCR. The sensitivity of the culture media was significantly higher for SCB (95.2 %, *p* = 0.004) than for the SBA (57.1 %). Despite the higher sensitivity for SCB (95.2 %) compared to CNA (76.0 %), the difference was not statistically significant (*p* = 0.078) (Table 2).

**Table 2:** Comparison of results for SBA, CNA, and the selective broth for detection of GBS

Culture medium	GBS positive samples (*n*)	Vaginal swabs (*n* =200)	
		GBS isolation rate (%)	Sensitivity (%)^a^
SBA	12	6.0	57.1
CNA	16	8.0	76.0
SCB	20	10.0	95.2
True positive GBS cultures	21	10.5	

^a^Sensitivity was calculated in comparison to true positive cultures of all media

The results of the direct latex agglutination test of positive cultures as determined by the SBA, CNA, and SCB methods (Table 3) revealed that 87.5 % of the samples yielded a positive result by latex method. Positive results by latex agglutination test from each culture method are as follows: SBA (83.3 % = 9/12), CNA (75 % = 12/16) and SCB (100 % = 20/20). Similar to the results of Study 1, the sensitivity, specificity, PPV, and NPV were 100 % for specimens with heavy or moderate growth density. For the low growth cultures, specificity and PPV remained at 100 %, but a decline in sensitivity and NPV was detected (Table 3).

**Table 3:** The results of latex agglutination test accompanied with the three culture methods

Culture medium	Level of GBS density (c.f.u. per swab)	No. of samples tested positive (*n*)	No. of samples positive for GBS (*n*)	Sensitivity (%)	Specificity (%)	PPV (%)	NPV (%)
SBA	High (>100)	3	3	100	100	100	100
	Moderate (50–100)	4	4	100	100	100	100
	Low (10–50)	3	5	60	100	100	71.4
	Little (<10)	0	0	/	/	/	/
CNA	High (>100)	3	3	100	100	100	100
	Moderate (50–100)	5	5	100	100	100	100
	Low (10–50)	4	7	57.1	100	100	70
	Little (<10)	0	1	0	100	100	50
SCB	High (>100)	15	15	100	100	100	100
	Moderate (50–100)	5	5	100	100	100	100
	Low (10–50)	0	0	/	/	/	/
	Little (<10)	0	0	/	/	/	/

Here, we also did the statistical power analysis. All individuals were excluded if they recently used antibiotics and showed symptomatic vaginal discharge or an acute illness. For the evaluation of different culture methods, we conducted statistical analysis by using the χ2 test with Yates’ correction with SPSS 19.0 software. The alpha level was set at 0.05, in which *p* < 0.05 was considered statistically significant. The detection rates for the vaginal swabs were 10.0 %, 8.0 %, and 6.0 % on SCB, CNA and SBA, respectively. The GBS isolation rates of SCB and CNA were higher than SBA. However, there were no statistically difference (*p* > 0.05) among them. The sensitivity of the culture media was significantly higher for SCB (95.2 %, *p* = 0.004) than for the SBA (57.1 %). Despite the higher sensitivity reported for SCB (95.2 %) compared to CNA (76.0 %) for the vaginal swabs, the difference was not statistically significant (*p* = 0.078). Thus, selective broth SCB provided the highest significant sensitivity for the detection of GBS among the three culture methods.

## Discussion

Research in the area of GBS infections is important for international perinatal medicine as pregnant women can be asymptomatic carriers [13, 14]. Studies indicate that use of antibiotic prophylaxis contributes to a significant decrease in the incidence of newborn GBS infection in the USA and UK [11, 17]. However, there are few reports on the detection methods for the identification of GBS in China.

We evaluated three culture mediums for the identification of GBS. Comparison of SBA, CNA and SCB methods revealed that SCB was the most reliable. The sensitivity of CNA was similar to previous reports [6, 9] as was the sensitivity of SCB [3]. SCB is more expensive than SBA or CNA but processing time is reduced. Our results provide support for the current recommendations proposed by CDC for GBS detection in pregnant women by the SCB method.

In addition, we investigated a direct latex method for rapid detection of GBS. Sensitivity and specificity of the latex agglutination test were previously reported as 85.7 % and 99.3 % [20]. In our study, we found the latex agglutination test results were dependent on the density of GBS colonization. We determined a GBS density greater than 50 c.f.u. tested positive by the latex method while below 50 c.f.u. resulted in a significant decrease in the sensitivity and negative predictive values. The advantage of using the latex agglutination method over the culture methods was ease of use and a reduction in turnaround time. Therefore, the latex method is very likely beneficial for pregnant women who are prone premature labor and require immediate GBS detection.

The study herein provides data on the effectiveness of detection methods and occurrences of GBS in China. We found that SCB provided the highest sensitivity for the detection of GBS among the three culture methods tested; however, the use of a direct latex agglutination method is an alternative option with reduced labor costs and turnaround time that can be implemented in critical situations.

## Abbreviations

GBS: Group B *Streptococcus*
SCB: Selective carrot broth
CNA: Columbia colistin-nalidixic agar
SBA: Sheep blood agar
PCR: Polymerase chain reaction
PPV: Positive predictive value
NPV: Negative predictive value
